# Psychiatric and Psychosocial Factors of Suicide Decedents and Survivor of Suicide Loss: Psychological Autopsy Study of Incheon City in South Korea

**DOI:** 10.3390/ijerph19137895

**Published:** 2022-06-27

**Authors:** Mi-Nam Bae, Seo-Eun Cho, Ju-Hyeon Ryu, Mi-Hwa Kim, Hye-Jin Jeon, Eun-Ji Shin, Seon-A Lee, Tae-Yeon Hwang, Seung-Gul Kang

**Affiliations:** 1 Incheon Metropolitan City Suicide Prevention Center, Incheon 21565, Korea; handsome82@ispc.or.kr (M.-N.B.); ddffdd@ispc.or.kr (J.-H.R.); mhwise@ispc.or.kr (M.-H.K.); hjjeon96@ispc.or.kr (H.-J.J.); 2 Department of Psychiatry, Gil Medical Center, Gachon University College of Medicine, Incheon 21565, Korea; arztin01@hanmail.net; 3 Korea Foundation for Suicide Prevention, Seoul 04533, Korea; ejshin@kfsp.org (E.-J.S.); seona08@kfsp.org (S.-A.L.)

**Keywords:** Korea, psychiatric disorder, psychological autopsy, psychosocial factors, suicide, survivor of suicide loss

## Abstract

In South Korea, the suicide rate is more than double the OECD average, and precise identification of the cause is required for suicide prevention. Psychological autopsy is used to reveal factors related to suicidal behavior; however, such studies are lacking in Korea. This study investigated the factors related to suicide using psychological autopsies in Incheon, a major city in Korea. In total, 46 cases were investigated using the Korea-Psychological Autopsy Checklist (K-PAC), and data on mental health conditions and psychosocial factors of suicide decedents and their families were analyzed. It was estimated that 87% of individuals of suicides had a mental health condition before death, but only 15.2% continued treatment or counseling. Most individuals who died of suicide showed warning signs before death, but only 19.6% of survivors of suicide loss noticed them. Mental health concerns before and after the death of the individual were also identified in more than half of their families. To prevent suicide, intensive and continuous treatment for psychiatric conditions and prompt recognition of active response to suicide warning signs are required. Care for the mental health of family members is also important.

## 1. Introduction

Death by suicide is not only an individual and family misfortune, but also a serious problem that causes enormous social and economic losses [1,2,3]. In 2020, Korea’s suicide rate was 25.7 per 100,000 people, which is the highest among OECD member countries [4]. According to a report on socioeconomic cost analysis of the five major causes of death published by the Health Insurance Policy Research Institute in 2015, suicide in Korea is the second-largest socioeconomic burden after cancer, and the economic burden of suicide is estimated at 6.47 trillion won (5.26 billion USD) per year [5].

Since suicide is considered preventable, it is important to identify risk factors for suicide, and the most effective method for this is to conduct a psychological autopsy [6]. Psychological autopsy is a procedure for estimating the cause of suicide by examining the psychological behaviors and changes of suicide decedents for a certain period before death through the statements of those who knew the individual and records related to them [7]. This process can contribute to the establishment of a suicide prevention policy by using the information collected through a psychological autopsy. Furthermore, this process has a positive effect on supporting healthy mourning of those impacted by suicide [8]. Many countries, including Finland, have conducted extensive psychological autopsies to closely analyze the root causes of national and regional suicide deaths. In Finland, various policy suggestions and services to reduce suicide deaths have been developed using the results of psychological autopsies [9].

The Korea Suicide Prevention Association performed the first psychological autopsy of seven individuals who died by suicide in Korea in 2009 using a systematic interview tool [10]. Since then, a number of institutions have performed psychological autopsies, but there are limitations such as a lack of standardization of research results due to the use of different psychological autopsy tools and difficulties due to the passive participation of those impacted by suicide [10,11]. To overcome these limitations, the Ministry of Health and Welfare established the Korea Psychological Autopsy Center (Korea Foundation for Suicide Prevention) in 2014 to identify the causes of suicide deaths in Korea and to establish evidence-based suicide prevention policies. The Korea Foundation for Suicide Prevention developed the Korea-Psychological Autopsy Checklist (K-PAC), a semi-structured interview tool, by extracting common questions through domestic and foreign literature reviews and selecting items appropriate to the Korean situation [12].

Previous foreign and Korean psychological autopsy studies have reported that mental health problems, including psychiatric disorders, are strongly related to suicide [13,14]. Among mental health disorders, the risk of suicide is high in individuals with major depression, substance abuse issues and schizophrenia; in particular, major depression is known to be the most suicide-related condition [15]. Approximately 2–15% of people with major depression die by suicide [16], and Cavanagh et al. reported in a psychological autopsy study of suicide deaths that approximately 60% of individuals who died by suicide experienced major depression or other mood disorders [17]. In a psychological autopsy study conducted in rural areas of Korea, 44% of individuals of suicides experienced depression [18]. Lee et al. [13] conducted a psychological autopsy using an abbreviated questionnaire tool, and confirmed that the frequency of mental health conditions in suicide deaths was as high as 44.1%, suggesting the importance of managing mental health issues. They also reported that economic problems were risk factors for suicide [13]. According to the results of a case-control study exploring suicide risk factors, a higher rate of mental illness was confirmed in the suicide group than in the control group [19]. It has been reported that the suicide rate is high in conditions in the following order: depressive disorder; alcohol and drug abuse; and schizophrenia [19]. As such, Korean psychological autopsy studies have been performed using significantly different psychological autopsy tools, and the amount and type of information collected has been significantly different. Therefore, more systematic research using a systematic interview tool is required.

The causes of suicide vary depending on country, region and period. The results of psychological autopsy research in different regions inevitably differ, depending on the characteristics of the target regions and populations [18,20,21,22,23,24]. Although the Korea Foundation for Suicide Prevention has promoted suicide prevention projects by publishing a national report based on the results of psychological autopsy interviews, it is difficult to understand the suicide characteristics in each region. Owing to these limitations, it was difficult to establish a suicide prevention policy tailored to the characteristics of the region. Consequently, this study was conducted to identify the characteristics of suicide in Incheon, where suicide prevention projects are underway. Incheon is the 5th largest city in Korea (2.95 million people, 5.7% of Korean population) as of January 2022, and also has Incheon International Airport, which is one of the best global airports. In 2020, the suicide rate per 100,000 people in Incheon was 26.5, which was higher than the Korean average of 25.7. While the overall suicide rate in Korea is falling, the rate of suicides in Incheon stood at 26.5 in 2020, in an increase from 25.9 in 2019. Therefore, this study investigating suicide in Incheon in detail is meaningful, and it will be possible to utilize these data for suicide prevention. By analyzing the results of 46 psychological autopsies of suicide deaths from 2016 to 2021 in the Incheon area, we performed an in-depth and comprehensive analysis of the psychiatric conditions and psychosocial factors of fatal suicides and individuals impacted by suicide in the Incheon area.

The purpose of this study was to identify: (1) the psychiatric conditions of individuals who died by suicide and their families through psychological autopsy; (2) major stressful events and suicide warning signs of those who died by suicide; and (3) information about fatal suicide cases such as demographic characteristics, suicide methods and places of discovery of the individuals.

## 2. Materials and Methods

### 2.1. Data and Subjects

From January 2016 to July 2021, the Korea Foundation for Suicide Prevention analyzed psychological autopsy interview data, collected using K-PAC, for consecutive cases of suicide deaths (46 cases in total) in Incheon [12]. The interviewees of psychological autopsy were those affected by the suicide and knew what happened to the suicide decedents during the six months prior to the suicide, and who were 19 years of age or older. The psychological autopsy was performed from 3 months to 3 years after the death of the deceased. All the survivors of suicide loss agreed to participate in a psychological autopsy interview, completed a written consent form to conduct a psychological autopsy, and were interviewed using K-PAC. Interviews were conducted by the principal interviewer commissioned by the Korea Foundation for Suicide Prevention and the assistant interviewer writing stenographic records. The principal interviewers received approval from the Qualification Management Subcommittee after completing the curriculum provided by experts (board-certified psychiatrists and mental health specialists) in the Korea Foundation for Suicide Prevention. This study was reviewed and approved by the Korean Association of National Institutional Review Board, designated by the Ministry of Health and Welfare (IRB, P01-202110-22-002).

### 2.2. Interview Method and Analysis Variables

To identify the characteristics of suicide deaths, we used K-PAC 2.1 and 3.0 [12], an evidence-based psychological autopsy interview tool developed by the Korea Foundation for Suicide Prevention through extensive literature review related to psychological autopsy [12]. The K-PAC is an investigation method to verify the cause of suicide by objectively examining the psychological and behavioral patterns and changes of suicide decedents through statements and records of the survivors of suicide loss, and provides the basis for establishing a framework plan for suicide prevention in Korea. It is used to help support healthy mourning of those impacted by suicide. During the interview, the principal interviewer first examines the psychological and emotional state of the those impacted by suicide while also conducting a semi-structured interview using the K-PAC tool. The interview usually takes 2–3 h. The psychological autopsy interview was recommended for 3 months after the deceased’s death to avoid overstimulating the psychological distress of the survivors of suicide loss, and to increase the objectivity of the interview. There were 23 principal and 40 assistant interviewers. The following strategies were implemented to minimize inconsistencies caused by the different interviewers: new interviewer training was conducted for 14 h, including theory and practice about psychological autopsy, and maintenance training was conducted for 8 h a year according to interviewer training. After each interview, a case review process was conducted by at least two raters other than the principal interviewer. In the case review process, the interview results of the principal interviewer were checked on an electronic case record sheet (e-CRF), and queries were presented accordingly. We attempted to minimize errors through feedback measures, such as re-examining the query and correcting it.

The variables used in this study were selected from the K-PAC interview data. Factors such as demographic information, suicide death information, stressors at the time of death, major stressful events during childhood and adolescence, warning signs before suicide death, presumed psychiatric conditions and suicide attempt history of suicide decedents and their families were included in the analysis. Table 1 presents the composition of the variables.

### 2.3. Analysis Method

In this study, IBM SPSS Statistics version 25.0 (IBM Corporation, Armonk, NY, USA) was used for the statistical analysis of the collected data, and frequency analysis was performed to understand the individuals’ characteristics.

## 3. Results

### 3.1. Demographic Characteristics of Suicide Decedents and the Suicide Case Information

Table 2 describes the demographic characteristics of the suicide decedents and information about the suicide events. Among the 46 suicide decedents, 33 (71.1%) were men, which was more than twice as many as the number of women. The most common age group was 35–49 years old (17 people, 37%). Among suicide decedents, 23 (50%) were high school graduates, 25 (54.3%) were employed and 25 (54.3%) were married. The most common place of death was home (27 people, 58.7%) and the most common method of death was hanging (28, 60.9%). Twenty-two (47.8%) among the first discoverers were family members. At the time of death, 22 (47.8%) of the deceased were under the influence of alcohol. Twenty-seven (58.7%) of the deceased had visited an institution for help and 14 (30.4%) had visited the Department of Psychiatry within 3 months before death.

### 3.2. Demographic Characteristics of Survivors of Suicide Loss

Table 3 describes the demographic characteristics of the survivors of suicide loss. The average age of the 58 survivors who participated in the psychological autopsy interview was 44.9 years (SD = 14.6), and 72.4% of them were women. If the bereaved family wished, counseling was conducted for two or more family members of the deceased. A majority of the interviewed survivors had a college degree or higher (46.6%), and 69.0% had a job. The average period from the time of death of the deceased to the date of the psychological autopsy interview of the survivors was 378.7 days (SD = 354.8), and the relationship between the interviewed survivors and the deceased was primarily the spouse (31%), followed by parents, children and siblings.

### 3.3. Stressors and Suicide Risk Factors

The stressful events and suicide-related behaviors of the suicide decedents are shown in Table 4. Twenty-three (50%) of the deceased had a major stressful event during childhood or adolescence, the most common events being disease or death of close persons (7 people, 15.2%). The most common stressful events immediately prior to the death were family and marital problems (37 people, 80.4%, multiple responses). Sixteen (34.8%) suicide decedents had a previous suicide attempt, and six (13%) had previously exhibited self-harm behaviors.

In the families of suicide decedents, there were 3 families (6.5%) in which at least one individual attempted suicide, and 18 families (39.1%) in which there was at least one individual who died by suicide. There were 5 (10.9%) suicide attempts and 8 (17.4%) suicide deaths among acquaintances. 

### 3.4. Psychiatric Conditions and Treatment History of Suicide Decedents

The presumptive psychiatric conditions of those who died by suicide are described in Table 5. It is estimated that 40 (87%) suicide decedents had psychiatric disorders before death, the most common conditions being depressive disorder (63%), schizophrenia spectrum and other psychotic disorders (10.9%) and substance use disorders (6.5%). Twenty-six (56.5%) suicide decedents had a history of receiving treatment or counseling for psychiatric problems, and only 7 (15.2%) maintained treatment or counseling until death. Among the 7 individuals, 4 (8.7%) received continuous treatment and 3 (6.5%) received intermittent treatment. The number of suicide decedents who had stopped treatment before the time of death was 10 (21.7%), of whom 5 were reluctant to receive psychiatric treatment and psychotropic medications.

### 3.5. Warning Signs of Suicide Decedents and Recognition of Survivors

Table 6 shows the suicide warning signs given by suicide decedents before death (multiple responses were allowed). The most common warning signs of the different categories of signs were: ‘often talking about suicide and death’ (verbal warning, 25 people, 54.3%), a change in eating status (behavioral warning, 31 people, 67.4%) and a change in emotional state (emotional warning, 33 people, 71.7%). Among the 46 suicide decedents, 43 (93.5%) had shown a warning sign before death; however, only 9 (19.6%) were recognized as a suicide warning sign by the those impacted by suicide before death.

### 3.6. Psychiatric Conditions and Treatment of Those Impacted by Suicide

Table 7 describes the psychiatric conditions of those impacted by suicide before and after the person’s death by suicide. There were 27 households suspected of having psychiatric problems among family members before the person’s death by suicide, and 46 family members were suspected of having psychiatric problems. Among them, 40 (87%) were presumed to have had a psychiatric condition, with depression being the most common (15 people, 32.6%). However, only 11 (23.9%) individuals had received treatment or counseling for psychiatric problems. 

After the death of the individual by suicide, 26 families had additional psychiatric problems and 37 family members were suspected of developing psychiatric problems. Twenty-three people (62.2%) were presumed to have psychiatric conditions, with depression being the most common. 

## 4. Discussion

In our psychological autopsy in Incheon, South Korea, we found that most suicide decedents had a mental health condition before death. In addition, it is presumed that many of their family members also had psychiatric conditions before the death of the individual by suicide and the survivors of suicide loss often developed psychiatric conditions after the death of the individual by suicide. Most suicide decedents had shown a warning sign before death, but only about a fifth were recognized as sending a suicide warning sign by those impacted by suicide before death. Among the major stressors of suicide, family and marital problems were the most common.

The results of this study showed that depressive disorder was the most common psychiatric disorder among suicide decedents, followed by schizophrenia and other psychotic disorders. According to a previous study, suicide risk in persons with psychiatric conditions is high, and the risk of suicide was reported to be even higher in persons with previous suicide attempts and psychiatric conditions [2,25,26,27,28]. In addition, studies have shown a decreased suicide risk when people with mental health conditions receive appropriate psychiatric treatment [29]. Only 26 (56.5%) of the suicide decedents in our study had received treatment or counseling for psychiatric problems, and only 4 (8.7%) maintained treatment or counseling until death. There are common false beliefs among the general public in Korea that psychiatric treatment can lead to disadvantages in employment, and that all psychotropic drugs have addiction potential and serious adverse effects, such as cognitive impairment [30,31]. To correct these beliefs, practical measures such as education, publicity, support for treatment costs for psychiatric patients and resolving the disadvantages of purchasing private insurance are necessary [32,33,34]. In Korea, the Mental Health Act was revised in 2016 to improve the human rights and autonomy of patients; however, psychiatric hospitalization procedures became very difficult without their own or their family’s consent, even if they were at risk of suicide or violence [35,36]. Therefore, it is necessary for the country to take responsibility for the treatment of individuals with psychiatric conditions by providing legal hospitalization or outpatient treatment support systems for persons with mental health concerns who are at a high risk of suicide/violence, as in the United States and other countries [37,38]. 

In this study, it was estimated that many survivors of suicide loss had mental health concerns before and after the death of a suicide decedent. In addition, there were many cases of deaths by suicide or attempted suicide among family members of those who died by suicide. This phenomenon is attributed to the shared genetic factors, stress factors and living environments among suicide decedents and their family members. After suicide deaths, survivors may experience a mixture of guilt, isolation, helplessness, despair, anger and resentment rather than other types of bereavement [39]. Survivors of suicide loss are at high risk of suicide, so they should be able to receive more intensive treatment and support services. In the past, support for survivors of suicide loss was limited to mental health-related areas [40], and more than half of survivors did not receive support as they did not know how to apply for the service [41]. A study conducted in Australia reported that community-based crisis interventions for survivors of suicide loss were practical and cost-effective [42]. Therefore, since 2019, the Ministry of Health and Welfare has been piloting a one-stop service support project for survivors of suicide loss, which is expected to reduce psychological and economic difficulties for survivors by supplying legal and administrative processing costs, children’s educational expenses and temporary housing support.

In this study, most of the deceased had shown a warning sign for suicide, but only one-fifth of the survivors recognized a warning sign from the deceased. In a previous Korean psychological autopsy study of 129 suicide deaths, 95.3% of suicide deaths were found to have had a suicide warning sign [43]. The Korean standardized Suicide Prevention Program on Intervention by gatekeepers was developed to train members of the community (“gatekeepers”) to detect suicide risk groups, identify and evaluate risk factors and refer individuals to experts. According to a study, after the educational program for suicide intervention, 95% of the gatekeepers listened to the reason for suicide, and 71.5% of them provided information regarding suicide prevention [44]. The common suicide warning signs in this study were frequent talking about death or suicide (verbal sign, 54.3%), changes in oral intake (behavioral sign, 67.4%) and emotional change (emotional sign, 71.7%). These results need to be actively used for suicide prevention projects to inform the public of important suicidal signs. The number of suicide attempts by family members was relatively low, considering other statistics (i.e., suicide death in decedents’ family). This may be cultural, with people being reluctant to accept the suicidal behavior of the family, or it might be because suicide decedents lacked support by others, thus few would be aware of any suicide attempts. In Korean society, a traditional culture based on Confucianism is formed, and there is a prejudice that suicide attempts are a personal matter and should be kept within the family. When a family member attempts suicide, some Koreans feel shame because of social prejudice and stigma, in addition to worry and anxiety toward suicide attempters. These prejudices can prevent them from seeking help related to mental illness and suicide. It is necessary to dispel social prejudice against suicide attempters and their families and create a social atmosphere where people can more openly talk about suicidal behaviors, crises and mental health so that those who are vulnerable to suicide can receive active support.

In this study, the most common stressors for suicide were those related to family and marital problems (37 people, 80.4%). This was followed by economic problems (29, 63%), occupational problems (25, 54.3%), interpersonal problems other than family members (12, 26.1%) and diseases and accidents (8, 21.7%). In past studies, economic problems and family stress were major risk factors [45]. In addition, there is a significant correlation in which suicidal thoughts become stronger as financial difficulties increase [45]. As economic difficulties are common stressors for suicide deaths, it is necessary to establish a stronger social safety net for vulnerable groups to cope with Korea’s socioeconomic polarization and welfare blind spots [46,47]. Quantitative analysis, such as statistical data alone, makes it difficult to grasp the risk factors for suicide and the personal meaning of people who took their own life. Since suicide is the result of a complex interaction between individuals and society, culture, the economy and the environment, a comprehensive and in-depth understanding of various risk factors is required to understand suicide. Cause-consequential thinking, which simply considers one or two risk factors to be the cause of suicide, oversimplifies suicidal behavior. A more eclectic approach is needed, focusing on individual cases rather than statistical quantitative analysis studies [48]. 

This study had some limitations. First, the number of cases (*n* = 46) of suicide-related deaths was relatively small. During the same period of suicide death in this study (from January 2015 to January 2021), a total of 4657 people died by suicide in Incheon, and in only 1% of these cases did the survivors participate in the psychological autopsy. However, the number of samples in this study was relatively large compared to previous psychological autopsy studies conducted in Korea. In addition, this study has added value compared to previous studies conducted in Korea because a detailed analysis of survivors of suicide losses, in addition to suicide descendants, was conducted. Second, not all cases were conducted by the same interviewer, or were conducted by several interviewers, so there might be inconsistencies due to multiple interviewers. However, to minimize inconsistencies and errors, all interviewers were educated and trained systematically and all cases were reviewed by different specialists. Third, since it took an average of one year to interview survivors after suicide, there is a possibility that the information may be less accurate.

Korea’s suicide rate in 2020 decreased slightly compared to 2010, but is still the highest among OECD member countries. Recognizing that suicide is not a personal but rather a national issue, the Korean government announced the “National Action Plan for Suicide Prevention” in January 2018, aiming to reduce the suicide rate to less than 20 per 100,000 people and the annual number of suicide deaths to less than 10,000. It is necessary to establish an effective suicide prevention policy based on projects with confirmed research results and active participation of local government organizations with policy-making authority is essential.

## 5. Conclusions

Mental health concerns were presumed to be among the majority of suicide deaths, with depressive disorder being the most common condition. It was estimated that a significant number of those impacted by suicide also had psychiatric concerns before and after the suicide death of a decedent. Most of the deceased showed a suicide warning sign before suicide, but only a small number of survivors recognized the warning sign. Since suicide is closely related to psychiatric illness and psychiatric illness is common in the families of suicide decedents, active treatment for people with psychiatric disorders such as depression and suicide prevention education for psychiatric patients’ families and mental health workers should be emphasized. A more systematic and large-scale study in this field is required in the future.

## Figures and Tables

**Table 1:** Selection of variables.

Classification	Variables for the Analysis among the K-PAC Interview Data
Demographic information	Age, sex, education, marital status, cohabitant and occupation
Information regarding suicide	Suicide method, place of death, suicide detector and discovery process, drunken state at time of death and previous suicidal behavior
Stressful event at the time of death	Occupational problem, economic problem, family problem, marital problem, breakup with lover, interpersonal problem, physical health problem, mental health problem
Developmental history of suicide decedents and information regarding those impacted by suicide	Negative childhood and adolescent experiences, previous psychiatric treatment of family and suicidal behavior of family and acquaintances
Suicidal warning signs	Verbal, behavioral and emotional changes

K-PAC, Korea-Psychological Autopsy Checklist.

**Table 2:** Demographic characteristics of suicide decedents and the suicide case information.

Demographic Characteristics and Suicide Case Information	Male (*n* = 33)	Female (*n* = 13)	Total (*n* = 46)
Age			
≤34 years old	7 (21.2)	6 (46.2)	13 (28.3)
35–49 years old	15 (45.5)	2 (15.4)	17 (37.0)
50–64 years old	7 (21.2)	4 (30.8)	11 (23.9)
≥65 years old	4 (12.1)	1 (7.7)	5 (10.9)
Level of education			
Graduated from high school	17 (51.5)	6 (46.2)	23 (50.0)
Graduated from a university/college	13 (39.4)	5 (38.5)	18 (39.1)
Graduated from middle school	3 (9.1)	2 (15.4)	5 (10.9)
Occupation, employed	19 (57.6)	6 (46.2)	25 (54.3)
Cohabitant	23 (69.7)	12 (92.3)	35 (76.1)
Marital status, married	20 (60.6)	5 (38.5)	25 (54.3)
Place of death			
Home	16 (48.5)	11 (84.6)	27 (58.7)
Public place	9 (27.3)	1 (7.7)	10 (21.7)
Others	8 (24.2)	1 (7.7)	9 (19.6)
Suicide method			
Hanging	20 (60.6)	8 (61.5)	28 (60.9)
Jumping	6 (18.2)	3 (23.1)	9 (19.6)
Gas poisoning	5 (15.2)	1 (7.7)	6 (13.0)
Person who first found suicide			
Family member	14 (42.4)	8 (61.5)	22 (47.8)
Stranger	8 (24.2)	1 (7.7)	9 (19.6)
Police officer or firefighter	5 (15.2)	2 (15.4)	7 (15.2)
Drunken state at time of death	16 (48.5)	6 (46.2)	22 (47.8)
Visit to institution for help within 3 months before death			
Visit to ask for help	19 (57.6)	8 (61.5)	27 (58.7)
Psychiatric clinic	8 (42.1)	6 (75.0)	14 (30.4)
Non-psychiatric clinic	7 (36.8)	1 (12.5)	8 (17.4)
Others	4 (21.1)	1 (12.5)	5 (10.9)

Data are presented as number (percentage). Frequency was calculated for either total deaths or the number of individuals in each sex.

**Table 3:** Demographic characteristics of survivors of suicide loss.

Demographic Characteristics of Survivors of Suicidal Loss	Total (*n* = 58)
Age (years)	44.9 (SD = 14.6)
Female sex	42 (72.4)
Level of education	
Graduated from a university/college	27 (46.6)
Graduated from high school	21 (36.2)
Graduated from middle school	4 (6.9)
Graduated from elementary school	4 (6.9)
Occupation, employed	40 (69.0)
Period from suicide death to interview with survivors of suicide loss (mean days)	378.7 (SD = 354.8)
Relationship with the deceased	
Spouse	18 (31.0)
Parents	16 (27.6)
Children	13 (22.4)
Siblings	7 (12.1)
Relatives	1 (1.7)
Colleagues	1 (1.7)

Data is presented as number (percentage) or average.

**Table 4:** Major stressful events and previous suicide-related behaviors of the suicide decedents.

Stressful Events and Suicidal Behavior	Male (*n* = 33)	Female (*n* = 13)	Total (*n* = 46)
Negative childhood and adolescent experiences			
Stressful event	16 (48.5)	7 (53.8)	23 (50.0)
Death and illness of close persons	6 (18.2)	1 (7.7)	7 (15.2)
Family problems	3 (9.1)	1 (7.7)	4 (8.7)
Abuse and sexual assault	3 (9.1)	0 (0.0)	3 (6.5)
Economic problem	1 (3.0)	1 (7.7)	2 (4.3)
Accidents and injuries	1 (3.0)	0 (0.0)	1 (2.2)
Others	3 (9.1)	4 (30.8)	7 (15.2)
Negative adulthood experiences			
Family or marital problem	27 (81.8)	10(76.9)	37 (80.4)
Economic problem	22 (66.7)	7 (53.8.)	29 (63.0)
Job problems	21 (63.6)	4 (30.8)	25 (54.3)
Interpersonal problems outside the family	7 (21.2)	5 (38.5)	12 (26.1)
Illness and accident	6 (18.2)	4 (30.8)	10 (21.7)
Love problems	3 (9.1)	0 (0.0)	3 (6.5)
Suicide-related behaviors of decedents			
Previous suicide attempts of suicide decedent	12 (36.4)	4 (30.8)	16 (34.8)
Previous self-harm behavior of suicide decedent	4 (12.1)	2 (15.4)	6 (13.0)
Suicidality of decedent’s family and acquaintances			
Suicide death in decedent’s family	14 (42.2)	4 (30.8)	18 (39.1)
Suicide attempt in decedent’s family	3 (9.1)	0 (0.0)	3 (6.5)
Suicide death of decedent’s acquaintances	6 (18.2)	2 (15.4)	8 (17.4)
Suicide attempts of decedent’s acquaintances	5 (15.2)	0 (0.0)	5 (10.9)

Data are presented as number (percentage). Multiple responses allowed. Frequency was calculated for either total deaths or the number of individuals in each sex.

**Table 5:** Presumptive psychiatric disorders and treatment history of suicide decedents.

Presumptive Psychiatric Conditions and Treatment	Male (*n* = 33)	Female (*n* = 13)	Total (*n* = 46)
Presumptive psychiatric conditions			
Depressive disorders	21 (63.6)	8 (61.5)	29 (63.0)
Schizophrenia spectrum and other psychotic disorders	3 (9.1)	2 (15.4)	5 (10.9)
Substance use disorders	3 (9.1)	0 (0.0)	3 (6.5)
Bipolar and related disorders	0 (0.0)	1 (7.7)	1 (2.2)
Trauma and stress-related disorders	1 (3.0)	0 (0.0)	1 (2.2)
Personality disorders	0 (0.0)	1 (7.7)	1 (2.2)
No diagnosis	2 (6.1)	0 (0.0)	2 (4.3)
Not evaluated by interviewer	3 (9.1)	1 (7.7)	4 (8.7)
Treatment history			
History of treatment/consultation	17 (51.5)	9 (69.2)	26 (56.5)
Continuous treatment	2 (6.1)	2 (15.4)	4 (8.7)
Intermittent treatment	0 (0.0)	3 (23.1)	3 (6.5)
Discontinued treatment	8 (24.2)	2 (15.4)	10 (21.7)

Data are presented as number (percentage). Frequency was calculated for either total deaths or the number of individuals in each sex.

**Table 6:** Warning signs of suicide decedents and recognition by survivors.

Suicide Warning Sign	Male (*n* = 33)	Female (*n* = 13)	Total (*n* = 46)
Suicide warning sign, total	31 (93.9)	12 (92.3)	43 (93.5)
Verbal signs			
Frequently talking about suicide or death	17 (51.5)	8 (61.5)	25 (54.3)
Self-deprecating words	15 (45.5)	4 (30.8)	19 (41.3)
Complaining of physical discomfort	11 (33.3)	3 (23.1)	14 (30.4)
Write the death-related contents in letters, diary, notebooks, etc.	5 (15.2)	4 (30.8)	9 (19.6)
Talk about people who died by suicide	3 (9.1)	3 (23.1)	6 (13.0)
Words longing for the afterlife	2 (6.1)	2 (15.4)	4 (8.7)
Questions about how to die by suicide	2 (6.1)	1 (7.7)	3 (6.5)
Behavioral signs			
Change in oral intake	22 (66.7)	9 (69.2)	31 (67.4)
Change in sleep status	20 (60.6)	8 (61.5)	28 (60.9)
Difficulty concentrating and making decisions	7 (21.2)	7 (53.8)	14 (30.4)
Self-harm or substance abuse	8 (24.2)	4 (30.8)	12 (26.1)
Efforts to improve bad interpersonal relationships and clean up personal problems	9 (27.3)	3 (23.1)	12 (26.1)
Clearance of belongings	6 (18.2)	3 (23.1)	9 (19.6)
Indifferent to one’s appearance	6 (18.2)	2 (15.4)	8 (17.4)
Unusual behavior	4 (12.1)	3 (23.1)	7 (15.2)
Making a plan for suicide	4 (12.1)	2 (15.4)	6 (13.0)
Giving valuable belongings to others	2 (6.1)	2 (15.4)	4 (8.7)
Emotional signs			
Change in emotional state	23 (69.7)	10 (76.9)	33 (71.7)
Lethargy, avoidance of people, loss of interest	18 (54.5)	8 (61.5)	26 (56.5)
Recognition of warning sign by suicide survivor before death	7 (21.2)	2 (15.4)	9 (19.6)
Action by the survivor in response to the suicide warning sign	7 (21.2)	1 (7.7)	8 (17.4)

Data are presented as number (percentage). Multiple responses allowed. Frequency was calculated for either total deaths or the number of individuals in each sex.

**Table 7:** Presumptive psychiatric disorders and treatment in the survivors of suicide loss.

Psychiatric Conditions and Treatment	Before the Suicide Death	After the Suicide Death
Presumptive psychiatric conditions		
Depressive disorders	15	17
Substance-related and addiction disorders	8	0
Sleep-wake disorders	3	2
Schizophrenia spectrum and other psychotic disorders	4	0
Neurocognitive disorders	4	0
Anxiety disorders	2	1
Neurodevelopmental disorders (intellectual disability)	2	0
Trauma and stress-related disorders	0	2
Personality disorders	1	0
Others	1	1
No diagnosis	2	9
Unevaluable	4	5
Treatment or counseling	11 (23.9)	35 (94.6)

Data are presented as number (percentage). Frequency of treatment or counseling was calculated for families with suspected mental health concerns.

## Data Availability

The datasets used in this study are publicly available at the “Korea Foundation for Suicide Prevention” at https://kfsp-datazoom.org (accessed on 5 August 2021).
